# Liquid Crystalline Nanoparticles Conjugated with Dexamethasone Prevent Cisplatin Ototoxicity In Vitro

**DOI:** 10.3390/ijms232314881

**Published:** 2022-11-28

**Authors:** Filippo Valente, Edi Simoni, Erica Gentilin, Alessandro Martini, Elisabetta Zanoletti, Gino Marioni, Piero Nicolai, Laura Astolfi

**Affiliations:** 1 Ear Science Institute Australia, Ear Sciences Centre, School of Medicine, University of Western Australia, Nedlands 6009, Australia; 2 Bioacoustics Research Laboratory, Department of Neuroscience-DNS, University of Padova, 35122 Padova, Italy; 3 Otolaryngology Section, Department of Neuroscience-DNS, University of Padova, 35122 Padova, Italy; 4 Interdepartmental Research Centre of International Auditory Processing Project in Venice (I-APPROVE), Department of Neurosciences, University of Padova, Santi Giovanni e Paolo Hospital, ULSS3 Serenissima, 30122 Venezia, Italy

**Keywords:** glycerol monooleate liquid crystalline nanoparticles, cubosome, hexosome, drug delivery system, OC-k3 cells, inner ear, glucocorticoid receptor

## Abstract

The conjugation of drugs with nanoparticles represents an innovative approach for controlled and targeted administration of therapeutic agents. Nanoparticle-based systems have been tested for the inner ear therapy, increasing the drug diffusion and being detected in all parts of the cochlea when locally applied near the round window. In this study, glycerol monooleate liquid crystalline NanoParticles were conjugated with Dexamethasone (NPD), a hydrophobic drug already used for inner ear treatments but defective in solubility and bioavailability. NPD has been tested in vitro in the cell line OC-k3, a model of sensory cells of the inner ear, and the therapeutic efficacy has been evaluated against cisplatin, a chemotherapeutic compound known to induce ototoxicity. After comparing the physical chemical characteristics of NPD to the equivalent naïve nanoparticles, an initial investigation was carried out into the nanoparticle’s uptake in OC-k3 cells, which takes place within a few hours of treatment without causing toxic damage up to a concentration of 50 µg/mL. The NPD delivered the dexamethasone inside the cells at a significantly increased rate compared to the equivalent free drug administration, increasing the half-life of the therapeutic compound within the cell. Concerning the co-treatment with cisplatin, the NPD significantly lowered the cisplatin cytotoxicity after 48 h of administration, preventing cell apoptosis. To confirm this result, also cell morphology, cell cycle and glucocorticoids receptor expression were investigated. In conclusion, the NPD system has thus preliminarily shown the potential to improve the therapeutic efficacy of treatments delivered in the inner ear and prevent drug-induced ototoxicity.

## 1. Introduction

The medical condition of unilateral or bilateral hearing impairment is defined as hearing loss: it is called conductive hearing loss when it is related to alterations of the external or middle ear and sensorineural hearing loss when it is related to the inner ear [1]. According to World Health Organization, about 466 million people worldwide have disabling hearing loss, referring to hearing loss greater than 40 decibels (dB) in the better hearing ear in adults and greater than 30 dB in the better hearing ear in children. One-fourth of people over 60 years of age are affected by disabling hearing loss caused by congenital or hereditary factors or induced by exogenous ones [2]. Exogenous hearing loss can be caused by numerous stress factors such as noise, trauma, infections and ototoxic effects of drugs. Among the drugs, there are substances which cause the loss or dysfunction of the auditory cells (hair cells and neurons), and the induced damage can be reversible or permanent [3].

Cisplatin ((SP-4-2)-diamminedichloroplatinum(II)) (CDDP) is a chemotherapeutic agent belonging to the class of platinum-containing anti-cancer drugs. It was the first platinum antitumor drug introduced in clinical practice and is still one of the most widely used agents to treat solid tumors affecting the head, neck, lungs, esophagus, stomach and genitourinary tract [4].

The structure of the CDDP includes a central platinum atom with four ligands, two amino groups and two hydrochloric groups; when the last two groups are disposed in cis conformation, the molecule exhibits chemotherapeutic activity. The cellular uptake of CDDP, although not fully understood, is thought to occur because of passive diffusion via transmembrane channels and facilitated diffusion processes [5]. Once inside the cells, the two chlorine atoms are substituted with two molecules of water, forming a monohydrate complex; this turns the CDDP into a very reactive and cytotoxic molecule, able to interact with the cellular proteins, the phospholipid membrane, the cytoskeleton microfilaments, RNA, nuclear and mitochondrial DNA. All these effects may induce cell death by apoptosis, which is at the base of CDDP’s antitumor role [6,7].

The clinical administration of CDDP induces severe dose-dependent side effects, including neurotoxicity, nephrotoxicity and ototoxicity [4]. A major limitation of the chemotherapy is also the development of a serious drug resistance of the cancer cells [8]. In detail, the CDDP ototoxicity is dose-dependent, affecting hearing and inducing tinnitus occurrence. However, there is a high variability of individual response due to metabolism, genetic factors, age, combination with other drugs and pre-existing hearing problems [9,10,11]. CDDP affects the cell population of the organ of Corti, the spiral ganglion and the stria vascularis, causing morphological alterations and cell death. The treatment with CDDP first induces the death of the hair cells of the cochlear basal turn and the neurons connected to them through damage to the cell DNA and the induction of apoptosis [12,13,14,15,16].

Several compounds have been tested in clinical practice and basic research against drug-induced ototoxicity, in order to reduce or mitigate cellular stress, reactive oxygen species (ROS) production and/or DNA damage [15,16,17,18]. Dexamethasone is a synthetic drug belonging to the family of the glucocorticoids and is used for a wide variety of clinical treatments. In inner ear therapies, steroids are mostly administered via the systemic route. However, with classic systemic drug administration, it is very difficult to reach the therapeutic concentration within the inner ear due to the inner ear blood barrier [19,20]. Moreover, the systemic use of prolonged high steroid doses can cause numerous side effects to other organs. Dexamethasone has been administrated with local inner ear applications in cases of autoimmune diseases, sudden hearing loss and currently for treatment of Meniere’s disease [21,22]. Dexamethasone has been shown to reduce the toxicity caused by noise or aminoglycoside administration; it has been reported that glucocorticoids may reduce cell stress and inner ear cell death rate in vivo in guinea pigs [23,24]. In the same animal model, other studies showed that dexamethasone was able to reduce CDDP ototoxicity in vivo, inducing overexpression of antioxidant enzymes [25]. The in vitro treatment with dexamethasone affects cellular pathways involved in the early state of the apoptotic process, including the expression of the mitogen-activated protein kinase (MAPK) pathway, with increased activation of Jun and p38 protein subfamilies [26].

The treatment of inner ear diseases through drug delivery faces numerous challenges such as the limited blood flow to the inner ear, the presence of physical barriers acting as a selective filter for drug transportation from the circulatory system to inner ear, the small size of the cochlea and its isolated anatomical position [27,28]. Research in local drug applications and medications has recently attracted interest because it can be a more effective treatment than the systemic one [29].

Novel drug delivery methods based on nanoparticles have been proposed as suitable drug delivery systems, which can provide sustained release and improves target specificity, drug half-life and diffusion [20,29]. Our group previously characterized in vitro the biocompatibility and internalization of liquid crystalline nanoparticles based on glycerol monooleate (NPs) in OC-k3 cells [30], as an inner ear cell, showing that the NPs are internalized after 4 h from the administration and induce a dose-dependent response in the cells and are well tolerated up to the concentration of 50µg/mL. Results showed that the use of NPs’ biocompatibility is affected by factors such as size, external charge, composition and embryonic origin of the selected cell lines [31,32,33]. The NPs are thermodynamically stable, biodegradable and have been previously localized in the cochlear basal turn of the guinea pigs when administered near the round window [34]. They are able to encapsulate both hydrophilic and hydrophobic compounds [35,36]; previous studies reported a release penetration ratio of dexamethasone loaded in NPs and administered in the in ocular tissue of rats as 3.5/4.5-fold higher than that of the drug alone [37]. In the literature, it is demonstrated that glycerol monooleate liquid crystalline nanoparticles are useful to sustain drug release and improve drug penetration [38,39].

In this work, we encapsulated dexamethasone into the glycerol monooleate nanoparticles and tested the efficacy of this drug delivery system in vitro in the OC-k3 cell line against CDDP-induced cytotoxicity. We investigated the protective effect action of dexamethasone NPs-mediated drug delivery to develop a model that can help to prevent the CDDP-induced apoptosis of inner ear cells and ototoxic effects.

## 2. Results

### 2.1. NPD Physical Chemical Characterization

**Figure 1:**
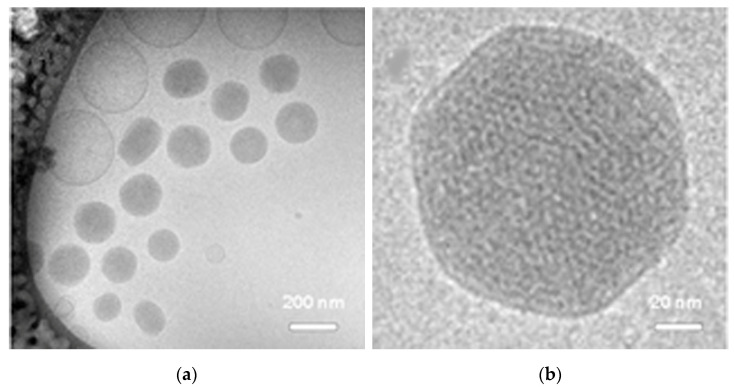
Structure of NPD by CRYO-TEM. (**a**). Image at low magnification. (**b**). Detail of an NPD. Scale bars as indicated.

Figure 1 reports the CRYO-TEM images of the produced glycerol monooleate nanoparticles functionalized with dexamethasone (NPD), and their physical chemical properties and stability in time are reported in Table 1. The NPD analyzed exhibited a rather roundish or hexagonal morphology [39] (Figure 1a,b). The NPD size ranged from 218 to 227 nm and they were highly homogeneous, with a low polydispersity index (PDI) (Table 1). NPD have a slightly negative surface charge (Z potential), due to the properties of the lipid used for particle synthesis, the glycerol monooleate. No significant variations were detected in physical and chemical parameters up to 60 days after preparation (Table 1).

### 2.2. NPD Cytotoxic and Protective Effects

**Figure 2:**
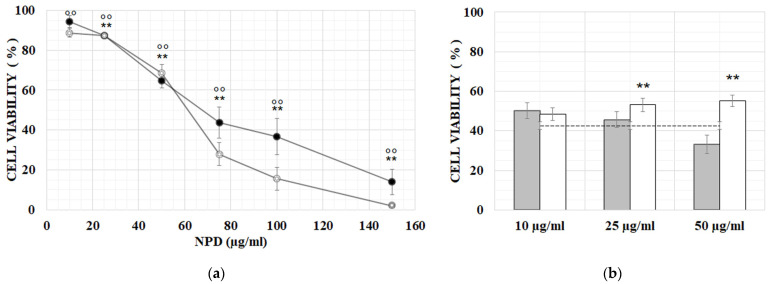
NPD cytotoxic and protective effects. (**a**) In vitro toxicity of NPD on OC-k3 cells, expressed as percentage of cell viability vs. NPD concentration (µg/mL) at 24 h (black dots) and 48 h (white dots), respectively. Bars represent the standard errors. The asterisk and circles indicate a significant difference in comparison to untreated controls at 24 and 48 h, respectively (** = *p* < 0.001; °° = *p* < 0.001). (**b**) Viability of OC-k3 cells pre-treated for 24 h with naïve NP (grey histograms) or NPD (white histograms) at the doses of 10, 25 and 50 µg/mL, then co-treated with 13 µM CDDP for 48 h (dotted line). Bars represent the standard errors. Asterisk indicates a significant difference in comparison to 13 µM CDDP (** = *p* < 0.01).

At 24 h, NPD were not significantly toxic up to 50 µg/mL: at this concentration, a 40% decrease in cell viability was detected in comparison to controls (*p* < 0.001) (Figure 2a). The toxicity of NPD increased in a significant way at higher concentrations (*p* < 0.001). At 48 h, from 50 µg/mL, the toxicity showed a sharper increase in comparison to 24 h (*p* < 0.001), inducing a 70% vitality decrease at the concentration of 75 µg/mL NPD. The NPD LD50 was 80 µg/mL and 66 µg/mL, respectively, at 24 and 48 h; meanwhile, the LD50 of the nanoparticles without dexamethasone was about 80 µg at both time points [33]. Based on these results, the protective effects were tested with the NPD doses of 10, 25 and 50 µg/mL, and the lower NPD concentrations were excluded because of the very low amount of the conjugated drug.

After 24 h of co-treatment, no significant recovery of cell viability was observed in comparison to CDDP alone. After 48 h of co-treatment, a recovery of cell viability was observed from 10 µg/mL NPD compared to the 13 µM CDDP alone, which became significant at 25 and 50 µg/mL NPD. The NPs without dexamethasone exerted lower protection effects, meaning, not significative, only at the concentration of 10 and 25 µg/mL (Figure 2b).

### 2.3. Dexamethasone Loading Efficiency

**Figure 3:**
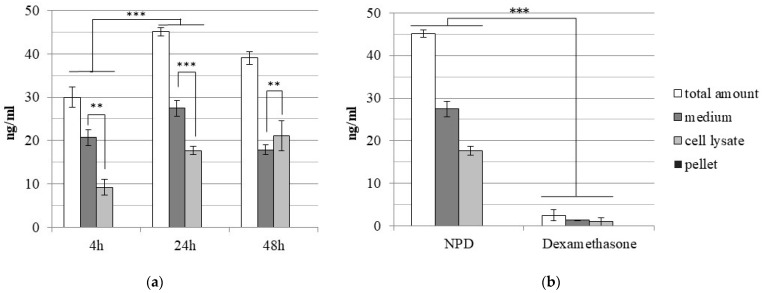
Amounts of dexamethasone (ng/mL) measured by ultra-high-performance liquid chromatography (UPLC) in extracts of OC-k3 cells. (**a**) OC-k3 cells treated for 4, 24 and 48 h with 50 µg/mL NPD. Asterisks indicate significant values between extracts treated with NPD at different times (** = *p* < 0.01; *** = *p* < 0.001). (**b**) OC-k3 treated for 24 h either with 50 µg/mL NPD (as in graph (**a**)) or with a corresponding amount of dexamethasone only for the same time. Asterisks indicate significant values between extracts treated with NPD and treated with dexamethasone only (*** = *p* < 0.001). Bars represent standard errors.

Starting from a given amount of NPD, the analyses by ultra-performance liquid chromatography (UPLC) allowed to obtain the concentration of the drug inside NPD. At the protective concentration of 50 µg/mL NPD, about 38 ng/mL of conjugated dexamethasone is released. The time-release profile of dexamethasone from 50 µg/mL NPD after 4, 24 and 48 h from treatment was also analyzed by UPLC. The results showed that the highest dexamethasone release occurred after 24 h (Figure 3a): at that time, the drug concentration in the culture medium or in the cell lysate was significantly higher (*p* < 0.001) in comparison to what was observed at 4 h. After 4 and 24 h of treatment, the highest dexamethasone concentration was detected in the culture medium and the amount was significantly higher (at 4 h, *p* < 0.01; at 24 h, *p* < 0.001) in comparison to what was detected in the cell lysate. At 48 h of treatment, the trend was reversed: the amount of dexamethasone was significantly higher in the cell lysate (*p* < 0.01) than in the culture medium. The amount of nanoparticle-conjugated drug released and internalized when cells were treated with NPD was analyzed by UPLC and compared to that in OC-k3 cells treated with a corresponding amount of drug alone at 24 h of treatment (Figure 3b). After 24 h, the culture medium, the cell lysate and the cellular pellet were analyzed in the treated cells’ lysate. The results showed that the amount of dexamethasone detected after 24 h in cells treated with NPD was significantly higher (*p* < 0.001) in comparison to those treated with the drug alone.

### 2.4. Effects of Treatment on the Cell Cycle

**Figure 4:**
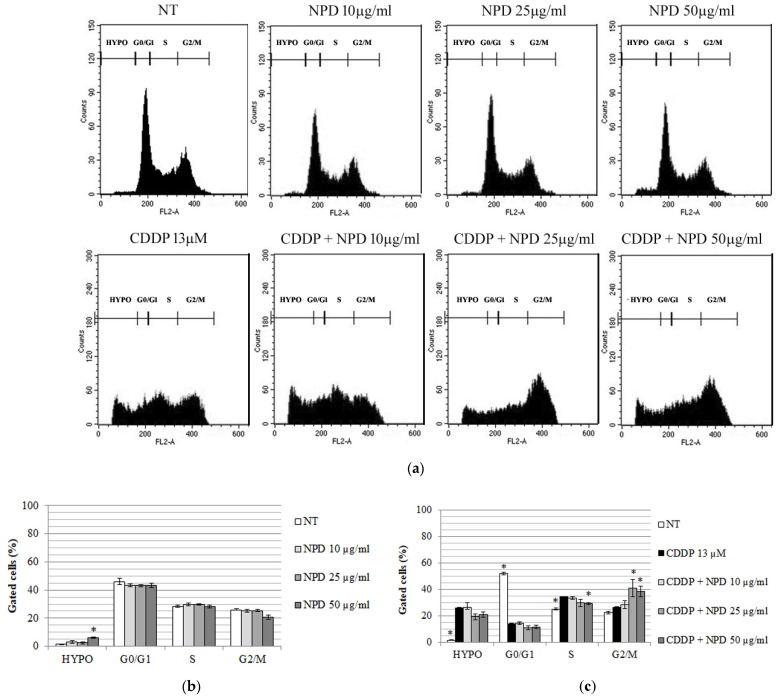
Analyses by flow cytometry of OC-k3 cells pre-treated for 24 h with NPD (10, 25 and 50 µg/mL), then co-treated with 13 µM CDDP for 48 h. (**a**). Number of cells (expressed as counts) in each phase of the cell cycle with and without CDDP co-treatment. For each experimental condition, about 20,000 events were analyzed. NT, untreated control. HYPO, hypodiploid cells. (**b**,**c**). Distribution of cells expressed as percentage of cells ± standard error in each cell cycle phase at 48 h with and without CDDP co-treatment, respectively. An asterisk indicates a significant difference in comparison to 13 µM CDDP (* = *p* < 0.05).

For the analyses of the cell cycle by flow cytometry on OC-k3 cells, the range of NPD concentrations examined was wide (10–50 µg/mL), but no significant variations were detected in the distribution of cell populations in the different phases of the cell cycle (Figure 4a,b). In detail, at 48 h, a slight dose-dependent increase in hypodiploid cells was detected that become significant with 50 µg/mL of NPD (Figure 4b).

The analysis of the cell cycle by flow cytometry in OC-k3 cells pre-treated for 24 h with NPD and then co-treated with CDDP for 48 h confirmed the dose- and time-dependent response of cells to co-treatments (Figure 4a,c). The CDDP induced a significant increase in the number of hypodiploid cells and of those in S, together with a decrease in those in G0/G1, and not significant differences were detected in the G2/M phase (Figure 4c). The co-treatment with NPD from 10 µg/mL onwards induced a not significant reduction in the number of hypodiploid cells. With increasing concentrations of NPD, a dose-dependent recovery of cells was observed in G2/M, which became highly significant at 25 and 50 µg/mL NPD (*p* < 0.01) at 48 h of co-treatment (Figure 4c).

### 2.5. Cell Morphology Analysis

**Figure 5:**
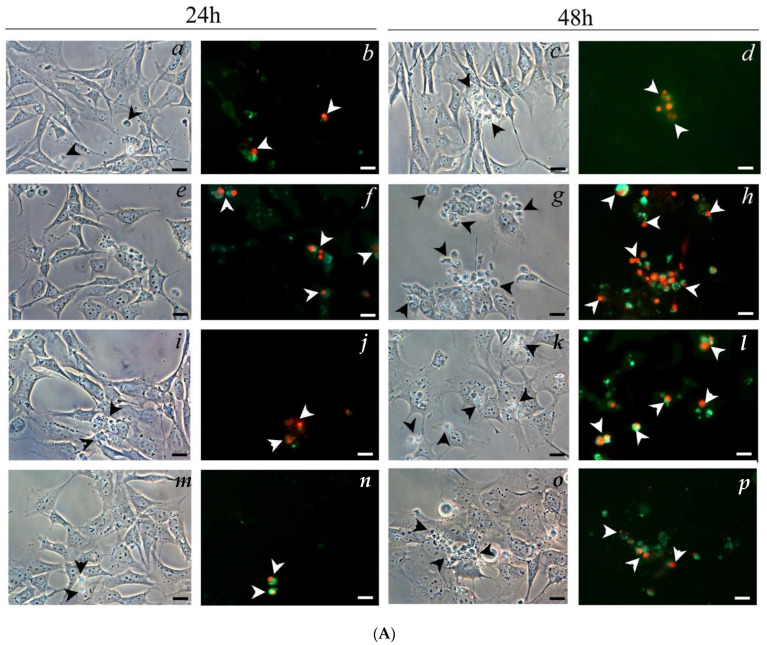

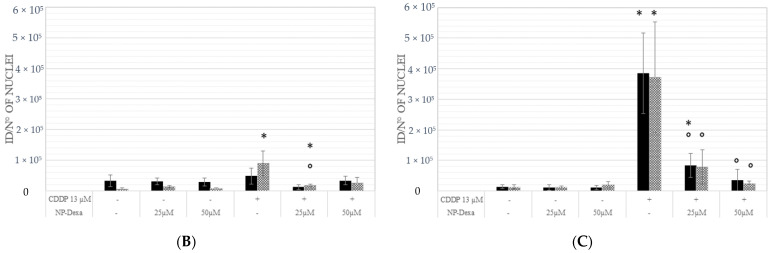
(**A**) Morphological investigations on OC-k3 cells. (**a**–**d**) Not treated cells. (**e**–**h**) Cells treated with 13 µM CDDP. (**i**–**l**) Cells co-treated with 25 µg/mL NPD and 13 µM CDDP. (**m**–**p**) Cells co-treated with 50 µg/mL NPD and 13 µM CDDP. (**a**,**c**,**e**,**g**,**i**,**k**,**m**,**o**) Phase contrast images. (**b**,**d**,**f**,**h**,**j**,**l**,**n**,**p**) Annexin V-FITC (green) and PI (red) staining. Arrowheads indicate cells in apoptosis. Scale bar 20 µm. (**B**,**C**) Quantification of apoptosis (annexin V, black bars) and necrosis (PI, diamond pattern bars) as mean ratio between the integrated density (ID) and the number of nuclei of three independent experiments after 24 h (**B**) and after 48 h (**C**), respectively. * = *p*< 0.05 vs. untreated control ° = *p* < 0.05 vs. cisplatin.

The morphological investigations with annexin V-FITC and PI on OC-k3 cells pre-treated for 24 h with NPD, then co-treated with CDDP and NPD for 24 and 48 h, revealed a marked time-dependent improvement of cell conditions in comparison to CDDP (Figure 5). After 24 h of treatment with 13 µM CDDP, OC-k3 cells appeared deformed and enlarged: many of them were positive to annexin V-FITC and PI (Figure 5A). At the same time of exposure in co-treatments with 25 and 50 µg/mL NPD, a decrease in the number of double-stained cells was detected (Figure 5B). At 48 h of treatment with 13 µM CDDP, the number of cells entering apoptosis increased. In co-treatments, the OC-k3 cells were more numerous and similar to untreated controls (Figure 5C). However, the majority of cells appeared elongated and adhered to the substrate: only the roundish ones appeared double stained, depending on increasing NPD concentrations (Figure 5A).

**Figure 6:**
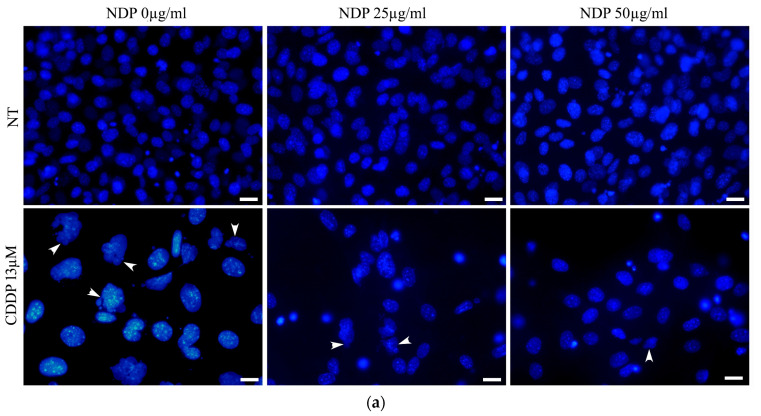

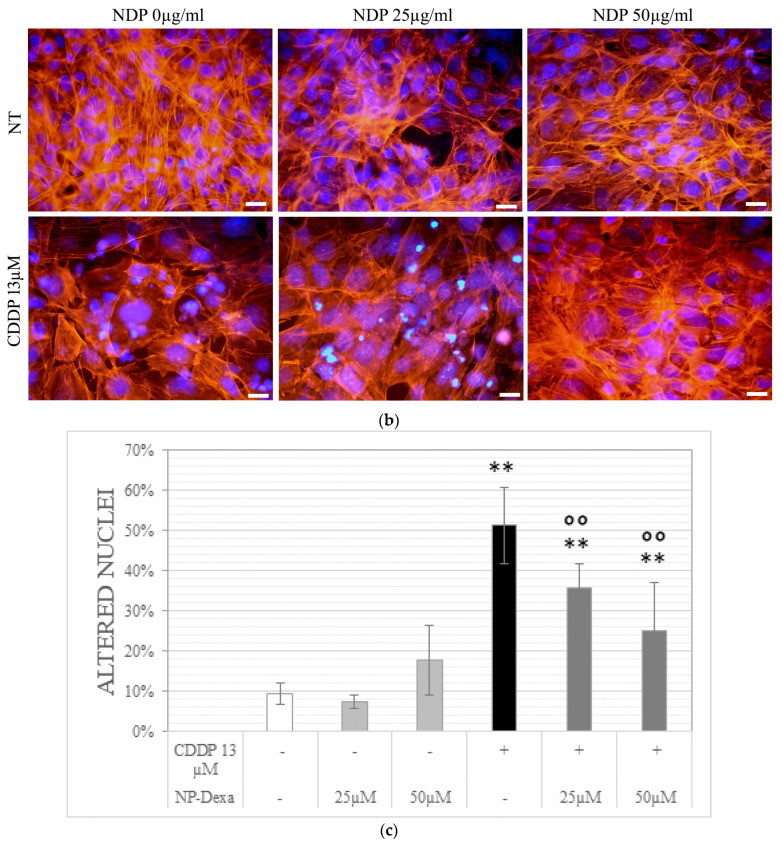
Morphological investigations on OC-k3 cells. (**a**). Nuclear staining with DAPI (blue); arrowheads indicate cells in apoptosis. (**b**). Cytoskeleton staining with phalloidin-TRITC (red) and DAPI (blue). (**c**). Percentage of altered nuclei. ** = significant difference in comparison to not treated cells, *p*-value < 0.01; °° = significant difference in comparison to 13 µM CDDP *p*-value < 0.01. Scale bar 20 µm.

The results obtained by morphological investigations with 4′,6-diamidino-2-phenylindole (DAPI) and phalloidin-TRITC on OC-k3 cells, pre-treated for 24 h with NPD, then co-treated with CDDP for 48 h, are reported in Figure 6. The treatment with 13 µM CDDP caused nuclear deformations in most cells, with very enlarged nuclei, and some multi-lobed nuclei were also visible (DAPI staining) (Figure 6a). These effects were confirmed by the phalloidin-TRITC staining, where only a few adhering cells were visible and those still attached to the substrate were swollen, with shorter cytoskeletal fibers, frayed cytoskeleton and scarce cytoplasm (Figure 6b). In co-treatments with NPD, the OC-k3 cells were more similar to untreated controls: the number of adhering cells was higher and most cells had physiological nuclei (Figure 6a,b). The percentage of altered nuclei significantly decreased, as compared to the CDDP treatment, in the presence of an increasing amount of NDP (Figure 6c).

### 2.6. Glucocorticoid Receptor Staining

**Figure 7:**
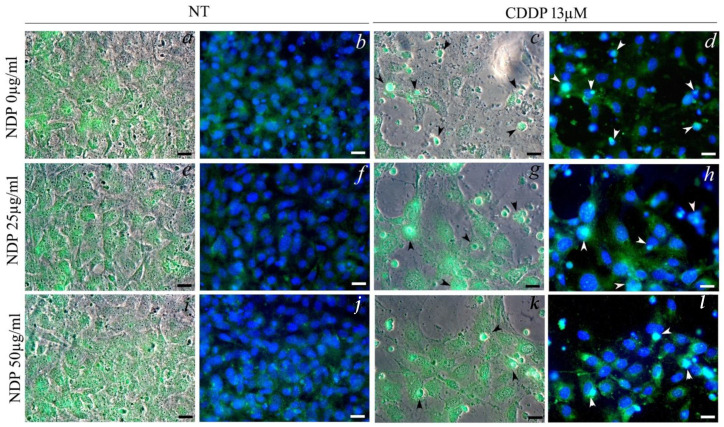
Morphological investigations on OC-k3 cells pre-treated for 24 h with NPD (25 and 50 µg/mL), then co-treated with 13 µM CDDP for 48 h. (**a**,**c**,**e**,**g**,**i**,**k**) Color combine: phase contrast and glucocorticoid receptor expression (green). (**b**,**d**,**f**,**h**,**j**,**l**) Color combine: nuclear staining with DAPI (blue) and glucocorticoid receptor expression (green). Arrowheads indicate cells with nuclear translocation of the GR. Scale bar 20 µm.

The glucocorticoid receptor staining showed a nuclear translocation in cells treated with the CDDP (22 ± 7% of cells). The NPD treatments did not alter the glucocorticoid receptor expression beside the release of dexamethasone. The percentage of nuclei expressing GR was zero for both doses tested, as in the untreated cells. In the co-treatments with 25 µg/mL NPD, it was found that 24 ± 16% of cells showed nuclear translocation, while the dexamethasone release from 50 µg/mL NPD was able to lower the nuclear translocation, which was observed only in 10 ± 4% of cells (Figure 7).

## 3. Discussion

The obtained results demonstrate that the administration of glycerol monooleate liquid crystalline nanoparticles loaded with dexamethasone (NPD) significantly reduces the cisplatin (CDDP) cytotoxicity in OC-k3 cells, derived from an organ of Corti of mice.

In this work, the morphological and physico-chemical characteristics of the NPD were altered by conjugating them with dexamethasone, compared to the drug-free NPs that we characterized in our previous work [33]. This includes the steric bulks’ appearance and variations of the surface charge. The NPD exhibited a ”hexagonal shape” compared to the NPs that showed a cubic structure (“cubosome”) with several lamellar structures interspersed among the cubic particles (as reported in [33]). It is well known that when lipophilic drugs are added to glycerol monooleate cubosomes, a transition from the cubic liquid crystalline phase to the reversed hexagonal one occurs [38,40]. Furthermore, it should be known that the most preferable model of drug release in the studies reported in the literature is the one obtained from the reversed hexagonal phase together with the cubic Micellar phase [40]. Nanoparticles showing a reversed hexagonal phase led to improved drug release; this transition is supposed due to packing stress in the hydrophobic regions of the hexagonal phase leading to a lower free energy level. The stability of the NPD formulation was confirmed up to 60 days, as well as surface charge, allowing structural cohesion and preventing auto-aggregation of NPs, as confirmed by polydispersion index results (Figure 1).

Dexamethasone can cause cellular stress and adverse effects when administered at very high dosages. For this reason, the choice of the amount to be administered is very delicate, especially if increased by a drug delivery system [41,42]. An ideal inner ear delivery system would reduce the dosage of dexamethasone administered while improving its diffusion, half-life and therapeutic effect on the site of interest.

In OC-k3 cells, the NPD induces a time-dependent toxic effect (Figure 2), similar to what is observed for the NPs [30]. It is interesting to notice that the most protective concentration of NPD in morphological and viability tests is 50 µg/mL, while the NPD alone cause dose-dependent toxicity. The NPD at high concentrations reduce the cell viability but, when administered ahead of cisplatin, protect the cells from cytotoxicity and cell death. This suggests that a higher amount of NPD induces a higher cellular stress, increasing cell response through the expression of genes related to apoptosis, whereas the high amount of internalized dexamethasone actively protects against cytotoxicity caused by CDDP and allows a higher cell survival rate.

Based on the evidence, further analyses were performed to quantify the amount of dexamethasone released from NPD and internalized in OC-k3 cells. The analysis by ultra-performance liquid chromatography (UPLC) of OC-k3 treated with 50 µg/mL NPD compared to cells treated with a corresponding amount of dexamethasone only (about 40 ng/mL) indicates that NPs significantly improve the cell uptake of dexamethasone, as previously shown in vivo [43]. By tracking the drug release over time, it was also possible to show that the highest amount of the compound was released in culture medium and inside cells after 24 h from treatment, in accordance with observations conducted with NPs conjugated to the fluorescent dye rhodamine B and with timing employed in co-treatment analyses [30]. Overall, these results clearly suggest that NPs induced a higher internalization of dexamethasone in cells in comparison to treatments with the compound alone. The dexamethasone uptake in cell lysate shows a significant increase from 4 h to 24 h and rises over time (until 48 h), as reported in previous studies, in which the highest amount of release was reached within 24 h, but its release profile depends on the drug solubility and its entrapment in the lipid bilayer [40]. Based on UPLC results, it is also likely that the conjugation to NPs may protect dexamethasone from degradation, increasing its half-life.

The NPD were tested at 10–50 µg/mL against different CDDP concentrations: they were able to protect against the highly cytotoxic CDDP concentration of 13 µM. Dexamethasone is indicated for chronic anti-inflammatory treatments, as it has been shown to become effective between 24 and 36 h after withdrawal [18,44,45]. In viability assays, NPD have not had appreciable recovery effects after 24 h, but after 48 h of co-treatment, a highly significant increase in cell viability was observed with 50 µg/mL NPD compared to 13 µM CDDP. Similar data were obtained in studies reported in the literature, where, in another inner ear cell line, HEI-OC1, the treatment with dexamethasone conjugated to other drug delivery systems (such as silk-polyethylene glycol hydrogel and solid lipid nanoparticles) lowered the cisplatin cytotoxicity mainly after 24 or 48 h of treatment [18,45]. Cisplatin treatment induces a drastic drop in G0/G1 phase cells, and instead of increasing the G2/M phase as expected, it seems to lead cells to cell death, as shown by the huge increase of cells in the hypodiploid phase (Figure 5). When cells have been co-treated with NPD, instead of inducing an arrest in G1/S as expected [46,47], given the protective effect obtained, it appears to induce an increase in G2/M phase cells. In previous studies, it has been defined that G2/M arrest is the consequence of a synergistic effect, for example, as demonstrated in testicular germ line tumors [46]. In fact, the cells that stop in G2/M are supposed to initiate the mitotic catastrophe, which leads to nuclei fragmentation and the formation of micronuclei [47]. Instead, after 48 h, the NPD treatment significantly decreases the number of cells in apoptosis or with nuclear alterations (Figure 6 and Figure 7). Dexamethasone is known to prevent cisplatin toxicity increasing the expression of antioxidant enzymes, preventing the apoptotic process and decreasing the proinflammatory cytokines [48,49]. In this regard, the involvement of the glucocorticoid receptor (GR) was recently deepened [50,51]. Cisplatin binds the GR at the C622 and recruits the receptor in the nucleus, and its activation induces the expression of the proteins involved in cell death, activating anti-apoptotic signaling upon cisplatin treatment [50]. The same effect was observed in OC-k3 cells, were cisplatin induced the nuclear translocation of GR, but, in the co-treatment, the NPD were able to prevent the nuclear translocation of the GR. Dexamethasone prevents the cisplatin-GR cascade binding the GR in another site; this biochemical mechanism was reported to explain the involvement of the GR in the platinum resistance [50,51,52], but in the meantime explains why dexamethasone is able to lower cisplatin cytotoxicity.

Dexamethasone is a glucocorticoid with anti-inflammatory and antioxidant properties widely used in hearing loss treatment but also in basic research [53,54]. However, it is known that systemic administration can cause numerous side effects due to steroids accumulation. For this reason, local administration, for example, through the tympanic membrane, is preferred for the inner ear [54,55]. In the recent literature, there is an increasing number of studies in which dexamethasone is spread through the electrode of cochlear implants [56,57,58]. The interest in this drug is also due to the fact that it has been shown that GR is widely expressed in most cochlear tissues [50,51,52]. This has prompted the study of how to produce tailor-made therapies specially to avoid compromising the delicate balance of cochlear homeostasis.

Encouraged by the results that report the protective effect of dexamethasone release against cisplatin in mice, several authors have tried to improve the efficiency given by the release of dexamethasone [59]. The literature shows that, in the field of nanomedicine applied to the treatment of deafness, there are many studies dealing with various nanoparticles and nanomaterials tested to improve the release of specific drugs for well-defined purposes, or to meet the biological characteristics of the target tissues and the chemical physical characteristics of the compounds [20,53,55].

Thus, various types of nanoparticles, such as polyethylene glycol-coated polylactic acid (PEG-PLA), were conjugated to dexamethasone and administered in rodents by different routes (bullostomy, round window and intraperitoneally). In all these studies, it was shown that the PEG-PLA–dexamethasone complex was able to reduce the ototoxicity of cisplatin, without giving complete protection [60,61,62]. It is known that, to cause appreciable ototoxic effects in rodents, it is necessary to treat them with a high amount of cisplatin; unfortunately, this causes a high mortality rate. In a previous study, we showed that the mortality rate was lowered by applying the cisplatin in more consecutive doses, obtaining the same damage of the single dose, and we were able to protect against damages with an antioxidant [63]. In conclusion, glycerol monooleate liquid crystalline nanoparticles, and in particular NPD, have preliminarily proven to be a good compromise to achieve a customized release not only in relation to dosage but also time. The next step of this work will be to treat animals with the NPD delivery system.

## 4. Materials and Methods

### 4.1. Nanoparticles Production and Characterization

Nanoparticles were produced by the research group of Dr. Andrea Fornara from the RISE Research Institutes of Sweden (Stockholm, Sweden). Glycerol monooleate (GMO RYLO MG 19 Pharma), purchased from Danisco A/S (Grindsted, Denmark), was composed of minimum 95% monoglycerides, maximum 10% diglycerides and maximum 2% triglycerides. The fatty acid content was minimum 88% oleoyl (C18:1). The triblock copolymer poly(ethylene oxide)(PEO)-poly(propylene oxide)(PPO)-poly(ethylene oxide)(PEO) (trade name Lutrol F127) was obtained from BASF (Lundwigshafen, Germany) and its approximate formula was PEO101PPO56PEO101 and had an average molecular weight of 12,600 g/mol. Dexamethasone (9α-fluoro-16α-methylprednisolone, D1756, powder, purity ≥ 98% for HPLC, molecular weight 392.46) was purchased from Sigma-Aldrich (St Louis, MO, USA).

Nanoparticles functionalized with dexamethasone (NPD) were prepared by dilution of an isotropic mixture of 5% wt lipid and 5% wt ethanol in F-127 1% solution. Incorporation of dexamethasone was performed, dissolving the active compounds in the lipid blend. The coarse samples were then homogenized five times for 5 min through a microfluidizer (MF) (Microfluidizer M-110s, Microfluidics Corp., Newton, MA, USA) equipped with a Z-type interaction chamber with a minimum passage of 100 μm. The fine liquid crystalline nanoparticle dispersions were heated in a steam sterilization autoclave Laboklav 25 (SHP Steriltechnik AG, Detzel Schloss/Satuelle, Germany) at 121 °C for 20 min. The total time for sterilization and cooling was about 1 h. Samples were then stored at 4 °C.

The dry content of the NPD dispersions was analyzed by freeze-drying a pre-weighed sample using an Epsilon 2–4 LSCplus freeze drier (Martin Christ Gefriertrocknungsanlagen GmbH, Osterode am Harz, Germany). The concentrations of active compounds in the NPs were assessed by diluting the freeze-dried particles in ethanol followed by analysis by UV spectrometry (Lambda 650, PerkinElmer instruments LLC, Shelton, CT, USA) at 278 nm for dexamethasone.

The size of the NPs was detected by a Zetasizer Zen3600 measurer (Malvern Instruments Ltd., Worcestershire, UK) using disposable cuvettes. The samples were diluted 10 times in purified water prior to analysis in disposable cuvettes and measured 3 times: each measurement consisted of 12 sub-runs. Refractive indices were set to 1.47 and 1.33, for lipids and water, respectively, and the temperature was kept at 25 °C during measurements. Data presented in this study correspond to the zeta-average particle size.

The zeta potential of particles with and without drug loading was determined by measuring the electrophoretic mobility with the Zetasizer Zen3600 (Malvern Instruments). Samples were diluted in purified water or 5 mM acetate buffer (pH 5.5) to a particle concentration of 0.5–2.0 mg/mL and analyzed in disposable measuring cells at 25 °C. Each sample was measured in triplicate.

The samples were prepared in a vitrification system with a controlled environment, kept at 25 °C and with humidity close to saturation to prevent evaporation from the sample during preparation. A droplet of each dispersion was placed onto a carbon-coated “holey” polymer film supported by a copper grid, gently blotted with a filter paper to from a thin liquid film (10–500 nm) and plunged into liquid ethane at −180 °C. The sample was then transferred into liquid nitrogen at −196 °C and then observed at Zeiss LIBRA-120 cryogenic transmission electron microscope (CRYO-TEM) (Carl Zeiss, Oberkochen, Germany) operating at 80 kV and with a working temperature below −160 °C to prevent the formation of ice crystals.

### 4.2. In Vitro Cell Culture

The OC-k3 cells [64] were cultured at 33 °C and 10% CO2 with complete culture medium DMEM (Dulbecco Modified Eagle’s Medium; Gibco BRL, Gaithersburg, MD, USA), 10% FBS (Fetal Bovine Serum; Gibco BRL), L-glutammine 2 mM (Gibco BRL), 50 U/mL Interferon γ (IFN, Genzyme, Cambridge, MA, USA) and 1% penicillin—streptomycin (Gibco BRL).

### 4.3. Cell Viability Assay

Cells were seeded on a 96-well plate, adding 5000 cells in 100 µL of medium per well. After 24 h, cells were treated for the NPD biocompatibility test with different concentrations of NPD from 10 to 150 µg/mL for 24 and 48 h. When performing co-treatment analyses, 13 µM CDDP (CDDP, Accord Healthcare, Milan, Italy) was added to cells 24 h after the treatment with NPD or nanoparticles without dexamethasone at the concentrations of 10, 25 and 50 µg/mL. The culture medium was replaced in order to avoid interferences between CDDP and NPD. The protection rate was detected after 24 and 48 h from the CDDP treatment. The control samples were treated only with CDDP or NPD or NPs without dexamethasone. Each concentration was tested at minimum in triplicate, and every experiment was repeated a minimum of three times against suitable controls.

Cell viability was detected according to the manufacturer’s protocol by the MTS assay (CellTiter 96^®^ AQueous MTS Reagent Powder, Promega, Woods Hollow Road, Madison, WI, USA). The absorbance value obtained for each sample was read after 3 h of incubation at 37 °C at 492 nm by the SIRIO plate reader (SEAC s.r.l, Florence, Italy). The absorbance values, expressed as optical density (OD), were normalized to the mean value of the respective non-treated sample, then expressed in percentage of viability.

### 4.4. Ultra-Performance Liquid Chromatography (UPLC)

The dexamethasone loading efficiency of NPD in OC-k3 was quantified at 4, 24 and 48 h after treatment with 50 µg/mL NPD. After each time point, the cell medium was collected, and then cells were harvested and lysed by a water solution of NaCl 300 mM, EDTA 5 mM and Triton X-100 0.5% and centrifugated at 15,000× *g* for 20 min in order to collect and separate the cell lysate from the cell pellet.

For UPLC analysis, each sample of cell medium, lysate and pellet was diluted in dichloromethane (100 µL, DCM) and the drug concentration was quantified in the filtrate by a UPLC system (Waters Corp., Milford, MA, USA) with an ultraviolet (UV) light. The system was equipped with a C18 column (1.7 µm, 2.1 mm × 150 mm; Model Acquity BEH300, Waters Corp.) operating at 40 °C. The mobile phase was acetonitrile/water 1:9 plus 0.03% of trifluoracetic acid (phase A) and acetonitrile/water 1:9 plus 0.03% of trifluoracetic acid (Phase B) at a flow rate of 500 µL/min. A gradient was used where the mobile phase composition was changed from 100% phase A to 39% (phase B) over a period of 3.4 min. The injection volume was 10 µL and the absorbance was measured at 292 nm (dexamethasone). The concentration of the drug expressed in ng/mL was measured in relation to a calibration curve and a correction factor compensating for adsorption to the filter.

### 4.5. Cell Cycle Analyses

Cells were seeded on a 6-well plate, adding 150,000 cells in 2 mL of medium per well. After co-treatments, cells were collected into a sterile test tube, centrifuged at 1500× *g* rpm for 5 min and the resulting pellet was washed with PBS 1X, while the supernatant was discarded. The pellet was fixed in 70% ethanol and washed twice with PBS 1X. The pellet was resuspended in a solution of PBS 1X, RNase 100 µg/mL and PI 50 µg/mL and left at room temperature for 1 h. Samples were analyzed in a BD FACSCalibur cytofluorimeter (BD Biosciences, San José, CA, USA). The Standard Optical Filter 585/42 nm (FL2) channel was used to discriminate the percentage of cells in each phase of the cell cycle and the values obtained were indicated as an area (FL2-A) in the results. Each experiment was performed at least in triplicate.

### 4.6. Cell Morphology Analysis

Cells co-treated for 24 and 48 h with nanoparticles and CDDP were washed twice in PBS 1X and then fixed with Glyo-Fixx™ for 30 min at room temperature (RT). Fixed cells were stained with an annexin V-fluorescein isothiocyanate (FITC) and propidium iodide (PI) kit (Abcam, Cambridge, UK) to evaluate the presence of apoptosis cells and were analyzed with the fluorescence microscope Nikon Eclipse TE2000-U model (Nikon, Milan, Italy), and the images were acquired using the Nis-Elements 3.0 Image Analysis System software (Nikon). The fluorescence intensity was measured as a ratio between the integrated density (ID) and the number of nuclei by using ImageJ software (https://imagej.nih.gov/ij/) (accessed on 1 January 2020). Each experiment was performed at least in triplicate.

To evaluate the state of the cytoskeleton and nucleus, cells were fixed in Glyofixx (Thermo Scientific, Waltham, MA, USA) for 30 min at room temperature, then permeabilized and stained with phalloidin—tetramethylrhodamine (TRITC) 1 µg/mL (Sigma Aldrich) for 2 h at room temperature. After being washed in PBS 1X, cells were stained with 1 µM 4′,6-diamidino-2-phenylindole (DAPI) (Sigma Aldrich) for 5 min in the dark. The slides were then mounted on a microscope slide with a drop of 20% glycerol for observation with the fluorescence microscope. The percentage of altered nuclei was measured for each treatment by analyzing the DAPI images. The ratio between the number of altered nuclei and the number of total nuclei was evaluated in six images for each experimental condition.

### 4.7. Glucocorticoid Receptor Staining

Fixed cells were rehydrated with 0.1% Tween in PBS for 15 min, then incubated in blocking buffer (Goat serum, Sigma-Aldrich) for 30 min at 37 °C. The samples were then stained with the anti-Glucocorticoid receptor (Santa Cruz Biotechnology, Dallas, TX, USA) 1:200 overnight at 4 °C, washed twice with PBS and then stained with Anti-Rabbit-FITC (Sigma-Aldrich) 1:200 for 45 min in the dark at RT. The slides were then mounted on a microscope slide with 20% glycerol for observation with the fluorescence microscope. Each experiment was performed at least in duplicate. The percentage of nuclei showing dexamethasone translocation was measured as the ratio between the number of nuclei expressing GR and the number of total nuclei; the analysis was performed evaluating four images for each experimental condition.

### 4.8. Statistical Analyses

Data were analyzed by the program GraphPad Prism 7 (GraphPad Software, Inc., La Jolla, CA, USA) to perform *t*-tests and one-way ANOVA tests to identify significant results.

## Figures and Tables

**Table 1:** GMO chemo-physical characteristics.

Time ^1^	Day 0	Day 30	Day 60
**Size ^2^**	218 ± 1.9	227 ± 1.2	226 ± 1.4
**PDI ^3^**	0.036	0.042	0.026
**Z p ^4^**	−20.4	−17.3	−20.5

^1^ Time interval expressed in days; ^2^ Size expressed in nm, mean ± standard deviation, ^3^ polydispersity index; ^4^ Z potential measured at 4 °C expressed in mV.

## References

[1] Fetoni A.R., Astolfi L. (2020). Cisplatin ototoxicity and role of antioxidant on its prevention. Hear. Balanc. Commun..

[2] Deafness and Hearing Loss. https://www.who.int/news-room/fact-sheets/detail/deafness-and-hearing-loss.

[3] Ciorba A., Astolfi L., Martini A. (2008). Otoprotection and inner ear regeneration. Audiol. Med..

[4] Astolfi L., Ghiselli S., Guaran V., Chicca M., Simoni E., Olivetto E., Lelli G., Martini A. (2013). Correlation of adverse effects of cisplatin administration in patients affected by solid tumours: A retrospective evaluation. Oncol. Rep..

[5] Ghosh S. (2019). Cisplatin: The first metal based anticancer drug. Bioorg. Chem..

[6] Gentilin E., Simoni E., Candito M., Cazzador D., Astolfi L. (2019). Cisplatin-induced ototoxicity: Updates on molecular targets. Trends Mol. Med..

[7] Tang Q., Wang X., Jin H., Mi Y., Liu L., Dong M., Chen Y., Zou Z. (2021). Cisplatin-induced ototoxicity: Updates on molecular mechanisms and otoprotective strategies. Eur. J. Pharm. Biopharm..

[8] Makovec T. (2019). Cisplatin and beyond: Molecular mechanisms of action and drug resistance development in cancer chemotherapy. Radiol. Oncol..

[9] Campbell K.C.M., Le Prell C.G. (2018). Drug-induced ototoxicity: Diagnosis and monitoring. Drug Saf..

[10] Pearson S.E., Taylor J., Patel P., Baguley D.M. (2019). Cancer survivors treated with platinum-based chemotherapy affected by ototoxicity and the impact on quality of life: A narrative synthesis systematic review. Int. J. Audiol..

[11] Rabiço-Costa D., Gil-da-Costa M.J., Barbosa J.P., Bom-Sucesso M., Spratley J. (2020). Platinum-drugs ototoxicity in pediatric patients with brain tumors: A 10-Year Review. J. Pediatr. Hematol. Oncol..

[12] van Ruijven M.W., de Groot J.C., Klis S.F., Smoorenburg G.F. (2005). The cochlear targets of cisplatin: An electrophysiological and morphological time-sequence study. Hear. Res..

[13] Previati M., Lanzoni I., Astolfi L., Fagioli F., Vecchiati G., Pagnoni A., Martini A., Capitani S. (2007). Cisplatin cytotoxicity in organ of Corti-derived immortalized cells. J. Cell. Biochem..

[14] Giari L., Dezfuli B.S., Astolfi L., Martini A. (2012). Ultrastructural effects of cisplatin on the inner ear and lateral line system of zebrafish (*Danio rerio*) larvae. J. Appl. Toxicol..

[15] Fetoni A.R., Eramo S.L., Paciello F., Rolesi R., Podda M.V., Troiani D., Paludetti G. (2014). Curcuma longa (curcumin) decreases in vivo cisplatin-induced ototoxicity through heme oxygenase-1 induction. Otol. Neurotol..

[16] Roldán-Fidalgo A., Martín Saldaña S., Trinidad A., Olmedilla-Alonso B., Rodríguez-Valiente A., García-Berrocal J.R., Ramírez-Camacho R. (2016). In vitro and in vivo effects of lutein against cisplatin-induced ototoxicity. Exp. Toxicol. Pathol..

[17] Astolfi L., Simoni E., Ciorba A., Martini A. (2008). In vitro protective effects of *Ginkgo biloba* against cisplatin toxicity in mouse cell line OCk3. Audiol. Med..

[18] Cervantes B., Arana L., Murillo-Cuesta S., Bruno M., Alkorta I., Varela-Nieto I. (2019). Solid lipid nanoparticles loaded with glucocorticoids protect auditory cells from cisplatin-induced ototoxicity. J. Clin. Med..

[19] Parnes L.S., Sun A.H., Freeman D.J. (1999). Corticosteroid pharmacokinetics in the inner ear fluids: An animal study followed by clinical application. Laryngoscope.

[20] Valente F., Astolfi L., Simoni E., Danti S., Franceschini V., Chicca M., Martini A. (2017). Nanoparticle drug delivery systems for inner ear therapy: An overview. J. Drug Deliv. Sci. Technol..

[21] Plontke S.K., Liebau A., Lehner E., Bethmann D., Mäder K., Rahne T. (2022). Safety and audiological outcome in a case series of tertiary therapy of sudden hearing loss with a biodegradable drug delivery implant for controlled release of dexamethasone to the inner ear. Front. Neurosci..

[22] Bae S.H., Lee J.M., Lee H.J., Na G., Kim S.H. (2021). Effect of dexamethasone combination with gentamicin in chemical labyrinthectomy on hearing preservation and vertigo control in patients with unilateral Meniere’s disease: A randomized controlled clinical trial. J. Clin. Med..

[23] Himeno C., Komeda M., Izumikawa M., Takemura K., Yagi M., Weiping Y., Doi T., Kuriyama H., Miller J.M., Yamashita T. (2002). Intra-cochlear administration of dexamethasone attenuates aminoglycoside ototoxicity in the guinea pig. Hear. Res..

[24] Takemura K., Komeda M., Yagi M., Himeno C., Izumikawa M., Doi T., Kuriyama H., Miller J.M., Yamashita T. (2004). Direct inner ear infusion of dexamethasone attenuates noise-induced trauma in guinea pig. Hear. Res..

[25] Daldal A., Odabasi O., Serbetcioglu B. (2007). The protective effect of intratympanic dexamethasone on cisplatin-induced ototoxicity in guinea pigs. Otolaryngol. Head Neck Surg..

[26] Dinh C.T., Chen S., Bas E., Dinh J., Goncalves S., Telischi F., Angeli S., Eshraghi A.A., Van De Water T. (2015). Dexamethasone protects against apoptotic cell death of cisplatin-exposed auditory hair cells in vitro. Otol. Neurotol..

[27] Juhn S.K., Rybak L.P. (1981). Labyrinthine barriers and cochlear homeostasis. Acta Otolaryngol..

[28] Inamura N., Salt A.N. (1992). Permeability changes of the blood-labyrinth barrier measured in vivo during experimental treatments. Hear. Res..

[29] Liu S.S., Yang R. (2022). Inner ear drug delivery for sensorineural hearing loss: Current challenges and opportunities. Front. Neurosci..

[30] Simoni E., Valente F., Boge L., Eriksson M., Gentilin E., Candito M., Cazzador D., Astolfi L. (2019). Biocompatibility of glycerol monooleate nanoparticles as tested on inner ear cells. Int. J. Pharm..

[31] Zook J.M., Maccuspie R.I., Locascio L.E., Halter M.D., Elliott J.T. (2011). Stable nanoparticle aggregates/agglomerates of different sizes and the effect of their size on hemolytic cytotoxicity. Nanotoxicology.

[32] Hinton T.M., Grusche F., Acharya D., Shukla R., Bansal V., Waddington L.J., Monaghan P., Muir B.W. (2014). Bicontinuous cubic phase nanoparticle lipid chemistry affects toxicity in cultured cells. Toxicol. Res..

[33] Valente F., Bysell H., Simoni E., Boge L., Eriksson M., Martini A., Astolfi L. (2018). Evaluation of toxicity of glycerol monooleate nanoparticles on PC12 cell line. Int. J. Pharm..

[34] Liu H., Chen S., Zhou Y., Che X., Bao Z., Li S., Xu J. (2013). The effect of surface charge of glycerol monooleate-based nanoparticles on the round window membrane permeability and cochlear distribution. J. Drug Target..

[35] Spicer P.T., Small W.B., Small W.B., Lynch M.L., Burns J.L. (2002). Dry powder precursors of cubic liquid crystalline nanoparticles (cubosomes). J. Nanopart. Res..

[36] Boge L., Bysell H., Ringstad L., Wennman D., Umerska A., Cassisa V., Eriksson J., Joly-Guillou M.L., Edwards K., Andersson M. (2016). Lipid-based liquid crystals as carriers for antimicrobial peptides: Phase behavior and antimicrobial effect. Langmuir.

[37] Gan L., Han S., Shen J., Zhu J., Zhu C., Zhang X., Gan Y. (2010). Self-assembled liquid crystalline nanoparticles as a novel ophthalmic delivery system for dexamethasone: Improving preocular retention and ocular bioavailability. Int. J. Pharm..

[38] Chang C.M., Bodmeier R. (1997). Swelling of and drug release from monoglyceride-based drug delivery systems. J. Pharm. Sci..

[39] Chen Y., Ma P., Gui S. (2014). Cubic and hexagonal liquid crystals as drug delivery systems. BioMed. Res. Int..

[40] Milak S., Zimmer A. (2015). Glycerol monooleate liquid crystalline phases used in drug delivery systems. Int. J. Pharm..

[41] Özel H.E., Özdoğan F., Gürgen S.G., Esen E., Genç S., Selçuk A. (2016). Comparison of the protective effects of intratympanic dexamethasone and methylprednisolone against cisplatin-induced ototoxicity. J. Laryngol. Otol..

[42] Shih C.P., Chen H.C., Lin Y.C., Chen H.K., Wang H., Kuo C.Y., Lin Y.Y., Wang C.H. (2019). Middle-ear dexamethasone delivery via ultrasound microbubbles attenuates noise-induced hearing loss. Laryngoscope.

[43] Chen G., Hou S.X., Hu P., Hu Q.H., Guo D.D., Xiao Y. (2008). In vitro dexamethasone release from nanoparticles and its pharmacokinetics in the inner ear after administration of the drug-loaded nanoparticles via the round window. Nan Fang Yi Ke Da Xue Xue Bao.

[44] Rhen T., Cidlowski J.A. (2005). Antiinflammatory action of glucocorticoids—New mechanisms for old drugs. N. Engl. J. Med..

[45] Chen Y., Gu J., Liu J., Tong L., Shi F., Wang X., Wang X., Yu D., Wu H. (2019). Dexamethasone-loaded injectable silk-polyethylene glycol hydrogel alleviates cisplatin-induced ototoxicity. Int. J. Nanomed..

[46] Mueller S., Schittenhelm M., Honecker F., Malenke E., Lauber K., Wesselborg S., Hartmann J.T., Bokemeyer C., Mayer F. (2006). Cell-cycle progression and response of germ cell tumors to cisplatin in vitro. Int. J. Oncol..

[47] Granada A.E., Jiménez A., Stewart-Ornstein J., Blüthgen N., Reber S., Jambhekar A., Lahav G. (2020). The effects of proliferation status and cell cycle phase on the responses of single cells to chemotherapy. Mol. Biol. Cell..

[48] Hazlitt R.A., Min J., Zuo J. (2018). Progress in the development of preventative drugs for cisplatin-induced hearing loss. J. Med. Chem..

[49] Wang X., Chen Y., Tao Y., Gao Y., Yu D., Wu H. (2018). A666-conjugated nanoparticles target prestin of outer hair cells preventing cisplatin-induced hearing loss. Int. J. Nanomed..

[50] Pan C., Kang J., Hwang J.S., Li J., Boese A.C., Wang X., Yang L., Boggon T.J., Chen G.Z., Saba N.F. (2021). Cisplatin-mediated activation of glucocorticoid receptor induces platinum resistance via MAST1. Nat. Commun..

[51] Ye R., Sun L., Peng J., Wu A., Chen X., Wen L., Bai C., Chen G. (2021). Design, synthesis, and biological evaluation of dexamethasone-salvianolic acid b conjugates and nanodrug delivery against cisplatin-induced hearing loss. J. Med. Chem..

[52] Lu Y.S., Yeh P.Y., Chuang S.E., Gao M., Kuo M.L., Cheng A.L. (2006). Glucocorticoids enhance cytotoxicity of cisplatin via suppression of NF-κB activation in the glucocorticoid receptor-rich human cervical carcinoma cell line SiHa. J. Endocrinol..

[53] Lin Q., Guo Q., Zhu M., Zhang J., Chen B., Wu T., Jiang W., Tang W. (2022). Application of nanomedicine in inner ear diseases. Front. Bioeng. Biotechnol..

[54] Gao Z., Schwieger J., Matin-Mann F., Behrens P., Lenarz T., Scheper V. (2021). Dexamethasone for inner ear therapy: Biocompatibility and bio-efficacy of different dexamethasone formulations in vitro. Biomolecules.

[55] Herr I., Ucur E., Herzer K., Okouoyo S., Ridder R., Krammer P.H., von Knebel Doeberitz M., Debatin K.M. (2003). Glucocorticoid cotreatment induces apoptosis resistance toward cancer therapy in carcinomas. Cancer Res..

[56] Astolfi L., Simoni E., Giarbini N., Giordano P., Pannella M., Hatzopoulos S., Martini A. (2016). Cochlear implant and inflammation reaction: Safety study of a new steroid-eluting electrode. Hear. Res..

[57] Scheper V., Hessler R., Hütten M., Wilk M., Jolly C., Lenarz T., Paasche G. (2017). Local inner ear application of dexamethasone in cochlear implant models is safe for auditory neurons and increases the neuroprotective effect of chronic electrical stimulation. PLoS ONE.

[58] Simoni E., Gentilin E., Candito M., Borile G., Romanato F., Chicca M., Nordio S., Aspidistria M., Martini A., Cazzador D. (2020). Immune response after cochlear implantation. Front. Neurol..

[59] Hill G.W., Morest D.K., Parham K. (2008). Cisplatin-induced ototoxicity: Effect of intratympanic dexamethasone injections. Otol. Neurotol..

[60] Martín-Saldaña S., Palao-Suay R., Aguilar M.R., Ramírez-Camacho R., San Román J. (2017). Polymeric nanoparticles loaded with dexamethasone or α-tocopheryl succinate to prevent cisplatin-induced ototoxicity. Acta Biomater..

[61] Sun C., Wang X., Zheng Z., Chen D., Wang X., Shi F., Yu D., Wu H. (2015). A single dose of dexamethasone encapsulated in polyethylene glycol-coated polylactic acid nanoparticles attenuates cisplatin-induced hearing loss following round window membrane administration. Int. J. Nanomed..

[62] Sun C., Wang X., Chen D., Lin X., Yu D., Wu H. (2016). Dexamethasone loaded nanoparticles exert protective effects against Cisplatin-induced hearing loss by systemic administration. Neurosci. Lett..

[63] Astolfi L., Simoni E., Valente F., Ghiselli S., Hatzopoulos S., Chicca M., Martini A. (2016). Coenzyme Q10 plus Multivitamin Treatment Prevents Cisplatin Ototoxicity in Rats. PLoS ONE.

[64] Kalinec F., Kalinec G., Boukhvalova M., Kachar B. (1999). Establishment and characterization of conditionally immortalized organ of Corti cell lines. Cell Biol. Int..

